# Plasma-Lyte 148 and Plasma-Lyte 148 + 5% glucose compatibility with commonly used critical care drugs

**DOI:** 10.1186/s40635-020-00311-5

**Published:** 2020-06-23

**Authors:** Sophie Hammond, Andrew Wignell, Paul Cooling, David A. Barrett, Patrick Davies

**Affiliations:** 1 School of Medicine, University of Nottingham, Nottingham, UK; 2 Paediatric Critical Care Unit, Nottingham Children’s Hospital, Derby Road, Nottingham, UK; 3 Pharmacy Department, Nottingham University Hospitals NHS Trust, Nottingham, UK; 4 Division of Advanced Materials and Healthcare Technologies, School of Pharmacy, University of Nottingham, Nottingham, UK

**Keywords:** Plasmalyte, Compatibility, Intensive care, Pharmacostability, Drug stability

## Abstract

**Purpose:**

Plasma-Lyte is a balanced, crystalloid intravenous fluid which has been shown to avoid the hyperchloremic metabolic acidosis associated with 0.9% sodium chloride. Data on physical, pH and chemical compatibility with other medicines are essential.

**Methods:**

The compatibility of adrenaline, dobutamine, dopamine, furosemide, midazolam, morphine and milrinone with Plasma-Lyte 148 (PLA) and Plasma-Lyte 148 with 5% glucose (PLA-G) was investigated. Control solutions were 0.9% sodium chloride and 5% glucose. Chemical stability was defined as < 5% concentration change with high-performance liquid chromatography (HPLC). Physical compatibility was assessed by checking for colour changes and precipitate formation. The pH of the admixtures was considered acceptable if between 5 and 9 at all time points. Six repeats were carried out for HPLC, 2 for physical compatibility checks and pH measurements, with all admixtures being tested at 0, 2 and 24 h after mixing.

**Results:**

All combinations were found to be chemically stable at 0, 2 and 24 h apart from furosemide with PLA-G at 24 h and midazolam with PLA or PLA-G at both 2 and 24 h. Only midazolam was physically incompatible when mixed with both Plasma-Lyte solutions. The pH remained stable in all admixtures, although not all pH values recorded were within the range of 5–9.

**Conclusion:**

All drugs excluding furosemide and midazolam were shown to be chemically, physically and pH stable at the tested concentrations when diluted with PLA and PLA-G.

## Introduction

Intravenous (IV) fluids play a fundamental role in both routine and emergency patient care across a wide variety of clinical settings. Many fluids have been developed over the years including 0.9% sodium chloride, Ringer’s solution, Hartmann’s solution and Plasma-Lyte 148, with each fluid having advantages and disadvantages. With such a wide variety of IV fluids available for clinicians to use, it is vital that the optimal product is chosen to optimise patient outcomes.

0.9% sodium chloride is the principal intravenous fluid in use, with nearly 10 million litres being infused intravenously each year in the UK [1]. However, it is considered to be physiologically less well-balanced as it differs significantly from the composition of extracellular fluid, particularly in relation to the concentrations of chloride, potassium and calcium present [2]. It also lacks the ability to buffer [2], and the supra-physiological levels of chloride can give rise to a hyperchloremic metabolic acidosis [3–5]; 0.9% sodium chloride has been shown to decrease pH, increase chloride levels and decrease base excess when compared to balanced solutions [6].

Hyperchloremia is also independently associated with poorer outcomes and mortality [7–11]. Hyperchloremia reduces renal blood flow, lowers glomerular filtration rate, causes afferent renal vasoconstriction and decreases urine output, which can subsequently lead to acute kidney injury requiring renal replacement therapy [5].

Balanced fluids have a composition reflecting that of extracellular fluid and are designed to minimise any metabolic disturbances. Plasma-Lyte 148 is an example, being an isotonic crystalloid solution which is physiologically similar to extracellular fluid composition in terms of both osmolarity (294 mOsm) and electrolyte composition [12, 13]. Acetate and gluconate are also present, acting as bicarbonate precursors which are metabolised in vivo [14].

Plasma-Lyte has been found to produce lower increases in chloride in diabetic ketoacidosis resuscitation, with quicker resolution of acidosis than occurs with saline [15]. Plasma-Lyte has also been shown to be superior to 0.9% sodium chloride in the rehydration of children suffering from acute gastroenteritis [16] as well as being associated with lower mortality, fewer post-operative infections and fewer complications when used after open abdominal surgery [17]. It has also led to improved acid-base status and less hyperchloremia at 24 h post injury in trauma patients, when compared to 0.9% sodium chloride [18]. Therefore, and also in accordance with the literature review by Weinberg et al., it does appear that the use of such solutions is preferable compared to 0.9% sodium chloride in improving physicochemical outcomes; however, more data is required [12, 19].

One key challenge associated with introducing Plasma-Lyte into routine clinical practice is the relative lack of compatibility data to support mixing this fluid with other medicines. This is especially pertinent in critical care environments and in younger patients due to the multitude of medicines used concurrently, difficulties in securing IV access and the high proportion of intravenous fluid load which comes from drug infusions. Incompatibility can result in degradation of medicines and inactivation, as well as precipitation.

Previous work investigating the stability of several drugs with Plasma-Lyte and Plasma-Lyte and 5% glucose, namely aminophylline, clonidine, fentanyl, furosemide, midazolam, morphine and salbutamol, found that excluding midazolam, these agents were both physically and chemically stable at ‘Y-site’ concentrations with both fluids [20]. Another study looking at a further 87 drugs found that 83 of these 87 drugs tested were compatible based on appearance and turbidity measurements following y-site mixing [21]. ‘Y-sites’ allow for the co-infusion of fluids and drugs, leading to a shorter contact time between maintenance fluids and drug infusions when compared to fluid and medicines being mixed in an infusion container and also meaning that drug concentrations are lower than the standard infusion concentrations at these sites (‘Y-site’ concentrations) [22].

Therefore, this study aimed to test the pharmaco-compatibility of Plasma-Lyte and Plasma-Lyte and 5% glucose with previous untested but frequently used intensive care drugs and to re-test some previously tested drugs, e.g. morphine and midazolam, at higher concentrations to determine if these fluids can be used to prepare infusion solutions of these agents.

## Methods

The chemical, physical and pH compatibility of adrenaline, dobutamine, dopamine, furosemide, midazolam, milrinone and morphine were analysed with four different intravenous fluid solutions. These are all frequently used drugs administered by continuous IV infusion on the Paediatric Intensive Care Unit (PICU) at the Queen’s Medical Centre, Nottingham. The IV solutions used were 0.9% sodium chloride (NS), 5% glucose (G5), Plasma-Lyte 148 (PLA) and Plasma-Lyte 148 with 5% glucose (PLA-G). The first two solutions are both standard diluents commonly used in a variety of clinical settings.

Measurements were recorded at 0 h (the time of mixing), 2 h and 24 h for all three variables. Measurement at 2 h allowed assessment of y-site compatibility as at standard infusion rates any infusions will have passed through a ‘Y-site’ within this time. By 24 h, it is expected that any clinically relevant drug changes would have occurred and compatibility at 24 h would also help support preparation of the drug infusions in the test fluids.

The drugs were mixed with each IV fluid to produce the concentrations shown in Table 1. We tested each mixture for colour, clarity and precipitation, pH, and concentration as per the NHS “Standard Protocol for Deriving and Assessment of Stability” [23].

**Table 1:** Concentrations tested for each drug, and the fluid ratios between the drug and Plasma-Lyte

Drug	Assayed concentration	Drug to Plasma-Lyte volume ratio
Adrenaline	250 μg/ml	1:3
Dobutamine	2 mg/ml	1:5.25
Dopamine	4 mg/ml	1:9
Furosemide	1 mg/ml	1:9
Midazolam	3 mg/ml	3:2
Milrinone	200 μg/ml	1:4
Morphine	1 mg/ml	1:9

Chemical compatibility was assessed by high-performance liquid chromatography (HPLC), using a Thermo Scientific UltiMate 3000 HPLC system, and a reversed-phase ‘ACE Excel 3 SuperC18’ column (150 × 2.1 mm) as the stationary phase. Analysis was performed using a diode array detector which measured UV absorbance at wavelengths appropriate for each drug. The concentration of the drug in the samples at each time point was calculated from the UV absorbance peak using a calibration curve. Each calibration curve had a minimum *R* value of 0.99 and was developed by using at least 5 solutions of varying known concentrations for each drug. The use of the calibration curve also ensured that consistent starting concentrations were achieved.

The aqueous mobile phases (used for all the drugs excluding midazolam) were water (aqueous) and acetonitrile (organic), both with the ion pairing agent heptafluorobutyric acid added at 0.1%. In the aqueous mobile phase, this led to a pH of approximately 2.15. For midazolam, an ammonium carbonate buffer and acetonitrile were used as the aqueous and organic mobile phases respectively due to the formation of a split peak when using water and acetonitrile. For this, 1.92 g of ammonium carbonate was dissolved in 1 l of water, to form a 20-mM solution with a pH of approximately 9.0.

Each combination of drug and fluid was repeated 6 times. The percentage change in concentration at 2 h and at 24 h was calculated for each sample and an average percentage change at each time point calculated for each drug/fluid combination from the six repeats. A clinically significant chemically incompatibility was defined as a greater than 5% change in concentration.

The pH measurements were recorded using a Fisherbrand Hydrus 3000 pH metre which was calibrated at pH 4.0, 7.0 and 10.0 before use. Two independent tests were performed for each pH measurement for each drug and fluid combination at 0 h, 2 h and 24 h.

The physical compatibility of the drugs and IV fluids was assessed by checking for colour changes and the formation of any precipitates against a standard monochrome background. Two repeats were completed for each drug and IV fluid combination.

All samples were stored within the laboratory with a controlled temperature of 22 °C ± 1 °C whilst being investigated.

## Results

### Chemical compatibility

Relative to starting concentration, the average change in concentration of adrenaline, dopamine, milrinone and morphine was less than ± 1.50%, for all four fluids at both 2 and 24 h.

For dobutamine, on average the concentration changed less than ± 1.00% with G5, less than ± 1.50% with PLA and PLA-G and less than ± 2.50% with NS. One repeat with PLA did decrease in concentration by 5.04% at 2 h. This was not significant at 24 h as the overall change was − 4.80%: a change of < 5.00% being defined as insignificant.

The average concentration changes for furosemide with G5, NS and PLA were all less than ± 1.00%. Upon further analysis of the results with G5, a single repeat showed a 5.12% decrease in concentration by 24 h which is a clinically significant change in concentration, but the average concentration change was not significant. However, with PLA-G, the average concentration change by 24 h was − 13.40% despite all concentration changes at 2 h being < 3.01%.

With midazolam, there were no clinically significant variations when mixed with either G5 or NS, with the average percentage changes being less than ± 1.50% with both fluids. However, PLA and PLA-G showed a concentration increase in all six repeats, with a mean of + 8.99% for PLA and + 8.10% for PLA-G by 2 h and therefore are not compatible with these fluids at these concentrations. By 24 h, for PLA, the concentration then fell so that the average concentration change was + 0.84%. However, when looking at the repeats individually, the change in concentration by 24 h ranges from − 14.59% to + 5.55%. With PLA-G, the concentration did not fall between 2 and 24 h as it did with PLA, and the average change in concentration by 24 h of midazolam with PLA-G was + 7.91%.

### pH investigations

A safe pH for peripheral infusion is any between 5.0 and 9.0 [24, 25]. Admixtures remaining in this pH range at all time points were as follows: milrinone with PLA, dobutamine with PLA and PLA-G, morphine with all fluids, dopamine with PLA and PLA-G, adrenaline with PLA and furosemide with all fluids. All other combinations of drug and fluid were outside of this range at at least one time point.

Of the four diluents, PLA is the most stable in terms of pH, with six of the seven drugs being of pH suitable for peripheral administration when mixed with this fluid. Midazolam was the exception in this case. NS and G5 showed identical results to each other, with five drugs suitable for central administration only, and two suitable to be given peripherally. With PLA-G admixtures, 3 of the 7 drugs were suitable for peripheral administration.

### Physical compatibility

Midazolam was the only therapeutic agent where physical compatibility was observed to be an issue. When initially mixed with PLA at 0 h, both repeats went cloudy white. By 2 h, a precipitate had also started to form on the sides of the vial. At 24 h, the admixtures were no longer cloudy; however, there was a fine white precipitate at the bottom and sides of the vial. With PLA-G, the solutions did not initially go cloudy as they did with PLA, but by 24 h, there was a fine white precipitate present on the bottom and edges of the vial. No precipitates formed when midazolam was mixed with either NS or G5. This indicates that midazolam is physically incompatible with both PLA and PLA-G at the concentrations tested. All other admixtures were physically compatible.

Table 2 gives an overview of the results.

**Table 2:**
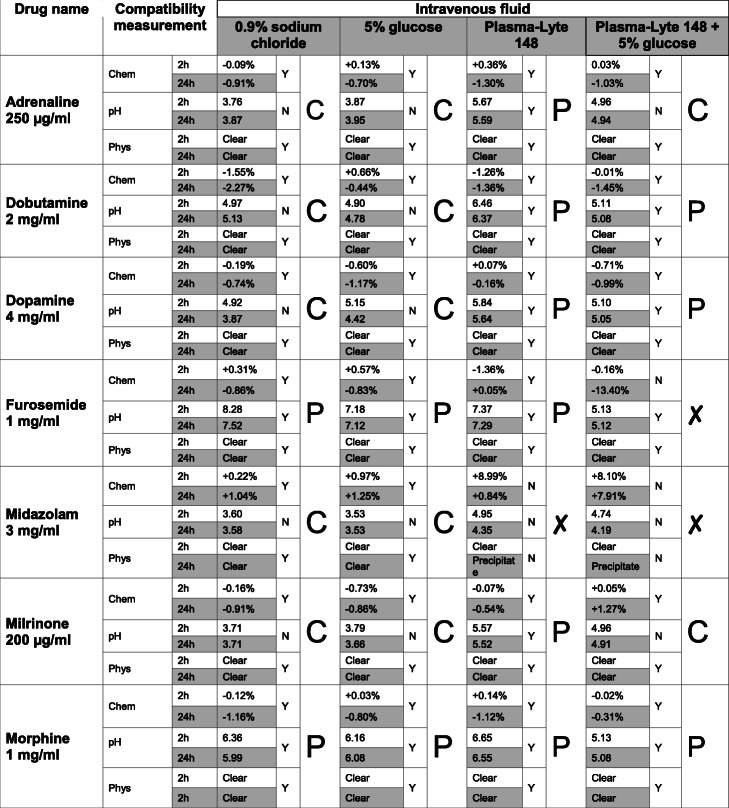
Overall compatibility of 8 frequently used infused therapeutic agents with 4 intravenous fluids

C indicates that the combination is suitable for central administration, P for peripheral administration and X that it is not compatible

## Discussion

We have provided new information on the chemical, pH and physical compatibilities of eight commonly used drugs when mixed with four commonly used fluids, adding to the compatibility knowledge base [20, 21].

### Chemical and physical compatibility

The only drug shown to be physically incompatibility with the Plasma-Lyte solutions was midazolam; it was also shown to be chemically incompatible; we believe due to the precipitant altering the measured concentration. Furosemide with PL-G was the other chemical incompatibility seen in this investigation. All other combinations of drugs and fluids at the tested concentrations are compatible.

The implications of this data for clinical practice will vary from setting to setting due to the differing needs of patients. Table 3 shows that children and adults often receive intravenous infusions at different concentrations, and therefore, the relationship between the concentrations tested in this research and those used in any given clinical setting will be variable. Furthermore, therapeutic agents can be directly infused with a fluid (correlating to standard infusion concentrations) or may be mixed with another fluid at a ‘Y-site’ to produce concentrations lower than the standard infusion concentrations [22, 26].

**Table 3:** Concentrations tested in relation to typical infusion concentrations and ‘Y-site’ concentrations used in children (10 kg) and adults and whether there is proven compatibility at these concentrations for Plasma-Lyte and Plasma-Lyte and 5% glucose admixtures

Drug	Concentration (μg/ml) as tested	Adults				Children (10 kg)			
		Standard infusion concentration (μg/ml)	Ratio of tested to infusion concentrations	Proven compatibility		Standard infusion concentration (μg/ml)	Ratio of tested to infusion concentrations	Proven compatibility	
				Infusion concentrations	Y site concentration			Infusion concentrations	Y site concentration
**Adrenaline**	250	80	3.13:1	✓	✓	60	4.17:1	✓	✓
**Dobutamine**	2000	5000	1:2.5	✗	✓	6000	1:3	✗	✓
**Dopamine**	4000	Not used				6000	1:1.5	✗	✓
**Furosemide**	1000	1000	1:1	✓*	✓*	10,000	1:10	✗	✓*
**Midazolam**	3000	5000	1:1.6	✗	✗	400	7.5:1	✗	✗
**Milrinone**	200	200	1:1	✓	✓	150	1.33:1	✓	✓
**Morphine**	1000	1000	1:1	✓	✓	200	5:1	✓	✓

*Only proven compatible with Plasma-Lyte

✗Not compatible at this concentration

All therapeutic agents that have shown compatibility with PLA and/or PLA-G in this study (all drugs excluding midazolam) therefore have proven compatibility at ‘Y-site’ concentrations often used in smaller children and adults (Table 3).

Furthermore, the concentrations tested for adrenaline, milrinone and morphine all directly relate to typical infusion concentrations used in a 10 kg child and adults, and furosemide too is compatible at typical infusion concentrations seen in adults (with PLA only). This means that PLA and PLA-G can be used as diluents for infusions of these medicines.

### pH investigations

Our study shows some surprising results. Midazolam is routinely given peripherally diluted either in NS or in G5; however, we found the pH of this mixture to be outwith the recommended range. Milrinone, also widely given peripherally when mixed with NS or G5, likewise is not suitable as the pH is too low. However, it is suitable for peripheral administration when diluted with PLA. Dobutamine is suitable for peripheral administration when mixed with either of the Plasma-Lyte solutions, but it is too acidic when mixed with NS or G5.

These findings suggest that, due to its inherent buffering properties, PLA is in general a safer diluent for peripheral administration than the standard diluents, i.e. NS and G5. This is likely to be especially important for drugs such as adrenaline and dopamine which intrinsically have extreme pHs and so are higher risk for both extravasation itself and significant tissue damage should this occur. Although these medicines are ordinarily given centrally, it is on occasion necessary to give them peripherally in the short term until central access can be secured. Not only would extravasation of an inotrope infusion in this situation run the risk of local tissue damage, it also means that such a critical medicine may not have the desired life-saving effect due to an interruption of intravenous delivery. Critically ill patients have a finite resource of peripheral veins accessible for access and administration of drugs, and these must be cared for judiciously.

## Conclusion

In conclusion, adrenaline, dobutamine, dopamine, milrinone and morphine are all chemically and physically compatible with PLA and PLA-G at the tested concentrations. Furosemide is chemically and physically stable with PLA, but not PLA-G at the tested concentration, and midazolam was only stable with the control solutions. In relation to midazolam, this study provides clear confirmation of results seen in previous work, which also suggested that midazolam is incompatible with PLA and PLA-G, albeit in a much more subtle manner [20]. pH investigations show that all admixtures were pH stable; however, not all admixtures are suitable for peripheral administration. PLA may be a more suitable diluent than NS or G5, particularly for drugs such as inotropes which have a high risk of extravasation when given peripherally.

By covering a range of ‘Y-site’ concentrations and direct infusion concentrations used in children (10 kg) and adults, this data will prove invaluable for practitioners looking to introduce PLA and PLA-G in their institutions.

## Data Availability

Data available on request.

## Abbreviations

PLA: Plasma-Lyte 148
PLA-G: Plasma-Lyte 148 with 5% glucose
HPLC: High-performance liquid chromatography
IV: Intravenous
UK: United Kingdom
SID: Strong ion difference
AKI: Acute kidney injury
PICU: Paediatric intensive care unit
NS: 0.9% normal saline
G5: 5% glucose
